# Effect of Thermosonication on Amino Acids, Phenolic Compounds, Sensory Properties and Microbial Quality in Freshly Squeezed Verjuice

**DOI:** 10.3390/foods12112167

**Published:** 2023-05-27

**Authors:** Başak Gökçe Çöl, Meryem Akhan, Burcu Çakmak Sancar, Melikenur Türkol, Seydi Yıkmış, Canan Hecer

**Affiliations:** 1 Department of Nutrition and Dietetics, İstanbul Gelisim University, Istanbul 34000, Turkey; bgcol@gelisim.edu.tr; 2 Department of Nutrition and Dietetics, İstanbul Esenyurt University, Istanbul 34510, Turkey; meryemakhan@esenyurt.edu.tr (M.A.); burcucakmak@esenyurt.edu.tr (B.Ç.S.); melikenurturkol@gmail.com (M.T.); cananhecer@esenyurt.edu.tr (C.H.); 3 Department of Food Technology, Tekirdag Namik Kemal University, Tekirdag 59830, Turkey

**Keywords:** thermosonication, verjuice, amino acids, phenolic compounds, RSM, antioxidant

## Abstract

Thermosonication is a process that can be used as an alternative to thermal pasteurization by combining mild temperature and ultrasound treatments. This study evaluated the effects of verjuice on the thermosonication process and its bioactive values modeled with the RSM (response surface method). The bioactive components of verjuice were found to increase with high predictive values. Additionally, the presence and amounts of 20 free amino acids in C-VJ (untreated verjuice), P-VJ (thermally pasteurized verjuice) and TS-VJ (thermosonicated verjuice) samples were investigated. Significant (*p* < 0.05) differences were detected among C-VJ, P-VJ and TS-VJ samples in all free amino acid values except methionine. Although 17 free amino acids were detected at various concentrations, glycine, taurine and cystine were not found in any samples. Thirteen phenolic filters in C-VJ, P-VJ and TS-VJ samples were also examined in this study. Eight phenolic donors with various abilities were detected in the C-VJ sample, along with nine phenolic acceptors in the P-VJ sample and eleven phenolic contents in the TS-VJ sample. The content of phenolic products in the TS-VJ sample increased by 37.5% compared to the C-VJ techniques and by 22.22% compared to the P-VJ techniques. Thermosonication did not significantly affect color and physiochemical values. Panelists generally appreciated the effects of thermosonication. It is concluded that the thermosonication process is a good alternative to thermal pasteurization. The results of this study provide essential data for future in vivo studies and show that the bioactive values of verjuice can be increased by using the thermosonication process.

## 1. Introduction

Verjuice (*Vitis vinifera* L.) is obtained by pressing unripe green grapes [1]. It is commonly added to traditional meals, vegetable salads and snacks to enhance flavor and is also recognized for its antimicrobial and antioxidant properties [2,3]. Verjuice has a high content of phenolic substances [4]. It is also used in the food industry as an acidifier and flavoring agent and has a self-protective system against pathogens such as *Escherichia coli* and *Salmonella typhimurium* [5].

Verjuice (unripe juice) has been found to contain various phenolic compounds, such as flavonoids and phenolic acids, which possess various health benefits. These compounds have been found to exhibit anti-inflammatory, antipathogenic, antiallergic, antidiabetic, antiviral, antimicrobial and antithrombotic effects on human health [6]. As a result, interest in the consumption of verjuice has been increasing due to its reported therapeutic benefits on human health.

The interest in non-thermal technologies for preserving fruit juices has increased due to consumers’ growing demand for high-quality and fresh food products. Thermal pasteurization, the most commonly used preservation technology, has adverse effects on fruit juices’ nutritional and sensory qualities. Therefore, alternative non-thermal technologies are appropriate [7,8,9].

The thermosonication method is a new food processing technique created by applying ultrasound with temperature control. It has been reported that thermosonication, which has recently gained popularity in food processing, provides microbial decontamination and protection while being reliable and environmentally friendly [10,11,12,13,14]. During the thermosonication process, it is essential to consider variables such as the temperature, pH, amplitude and processing time of the product to ensure the effectiveness of the process.

The thermosonication process has been applied in various studies on different types of fruit juices, including anthocyanin-enriched tomato juice [15], carrot (*Daucus carota*) juice [16], orange juice, whey juice [17], pineapple juice, cranberry juice, grape juice [18], prune (*Spondias mombin* L.) juice [19], beetroot (*Beta vulgaris* L.) juice [20] and fruit (*Haematocarpus validus*) juice [13]. However, it has been observed that the thermosonication process may cause changes in nutritional and sensory properties.

Thermosonication has an impact on the quality of juices and consumer consumption. When compared with thermal treatments, thermosonication results in less flavor loss; significant energy savings; the preservation of nutritional and sensory characteristics; increased homogeneity; the promotion of color lightness; increased juice extraction; the retention of ascorbic acid in the juice; inactivation of the polyphenoloxidase enzyme, which causes enzymatic browning reactions; and increased microbial inactivation, and it is thought that it can be used to make high-quality fruit juices without additives and preservatives. This method is particularly relevant to consumers who value freshness in products with a longer shelf life [21,22,23,24].

Process modeling approaches, such as the response surface methodology (RSM), are necessary to optimize conditions effectively. A literature review revealed numerous studies that have extensively investigated the thermosonication process and the optimization of fruit and vegetable juices. However, no study was found that compares the effect of the thermosonication process on the amino acids, phenolic compounds, sensory properties and microbial quality of verjuice using the RSM optimization technique. Therefore, this study aims to investigate the impact of thermosonication on the amino acids, phenolic compounds, sensory properties and physicochemical and microbial quality in verjuice.

## 2. Materials and Methods

Verjuices (*Vitis vinifera* L.) were collected as raw grapes from the Balıkesir region of Turkey in June 2022. The collected fruits were washed with clean water. The verjuice was squeezed using a home juicer (SJ-3143, Sinbo, Istanbul, Turkey) and filtered through a double-layered sterilized muslin cloth to remove coarse particles and impurities from the juice. Freshly squeezed verjuice was transferred to 100 mL sterile bottles. Freshly squeezed verjuice without treatment was called the untreated sample (control verjuice, C-VJ). Samples were stored at −18 ± 1 °C until analysis. Tests were performed three times.

### 2.1. Thermal Pasteurization and Thermosonication Processing

Thermal pasteurization treatment (P-VJ) was conducted in a water bath (Wisd-Model WUC-D06H, Daihan, Wonju, Korea). The sterile bottle was pasteurized at 75 °C for 15 s. After the P-VJ treatment, the juice was cooled down in an ice bath and manually packed into sterile bottles. For thermosonication treatment, 100 mL of freshly squeezed verjuice was processed using a 200 W ultrasonic processor (Model UW 100, BANDELIN electronic GmbH & Co. KG, Berlin, Germany) at a frequency of 26 kHz. The studied TS parameters included temperature (30, 35, 40, 45 and 50 °C ), processing duration (2, 4, 6, 8 and 10 min) and amplitude (40%, 45%, 50%, 55% and 60%) in constant mode. An ice-water bath was used to prevent overheating during ultrasound processing. All the treatments were performed in the dark during an ultrasound to avoid any possible interference of light. After thermosonication treatment, the verjuice samples were immediately cooled in an ice bath. Samples were stored at −18 ± 1 °C until analysis. Verjuice samples were named TS-VJ (thermosonication-treated verjuice) after optimization. Tests were performed three times.

### 2.2. Modelling Procedure for Response Surface Methodology

The response surface method (RSM) was used to understand the effect of thermosonication treatments on the bioactive components in the freshly squeezed verjuice. Temperature (X_1_, 30–50 °C ), time (X_2_, 2–10 min) and amplitude (X_3_, 40–60%) were independent factors, and total phenolic content (mg GAE/100 mL), total flavonoid content (mg CE/100 mL), DPPH (% inhibition) and CUPRAC (% inhibition) were response variables. For RSM, the Central Composite Design (CCD) was implemented using Minitab software (version 19, Minitab software, State College, PA, USA) to optimize the thermosonication processing of freshly squeezed verjuice. A five-level, three-factor, two-replicates experimental design was created. There were 20 trial points for optimization. The following quadratic–polynomial formula was used to create the equation models:

(1) $$y=\beta_{0}+\sum_{i=1}^{3}\beta_{i}X_{i}+\sum_{i=1}^{3}\beta_{ii}X_{i}^{2}+\sum_{\begin{array}{c} i=1\\ i<j \end{array}}^{3}\sum_{j=1}^{3}\beta_{ij}X_{i}X_{j}$$

The definition of this formula is as follows: the dependent variable (y); the intercept term (β_0_); the first order (linear) equation coefficient (β_i_); the quadratic equation coefficient (β_ii_); the two-factor cross-interaction coefficient (β_ij_); and the independent variables (X_i_ and X_j_).

### 2.3. Determination of Bioactive Compounds

TPC (Total Phenolic Content) was determined using the Folin–Ciocalteu method as described by Singleton and Rossi (1965) with a spectrophotometer (Spectrum Instrument, SP-UV/VIS-300SRB, Victoria, Australia) [25]. TPC was determined in triplicate for each treatment, sampling day and replicate, and the results are expressed as mg of gallic acid equivalents per 100 mL. Total flavonoid content (TFC) was calculated colorimetrically with a UV spectrophotometer (Spectrum Instrument, SP-UV/VIS-300SRB, Australia) according to the method applied by Zhishen et al. (1999) [26]. Antioxidant activity was assessed using two different methods: the scavenger 2,2-diphenyl-1-picrylhydrazyl (DPPH) radical and cupric ion reducing antioxidant capacity (CUPRAC) following the methodologies previously described by Grajeda-Iglesias et al. (2016) and Apak et al. (2006), respectively [27,28]. Analyses were performed in triplicate.

### 2.4. Determination of Phenolic Compounds

Phenolic compounds were analyzed using an Agilent 1260 Infinity chromatograph with a diode array detector (DAD). The chromatographic procedure was as described by Portu et al. (2016), using a C-18 Age Generix column (250 × 4.6 mm; 5 µm packing; Waldbronn, Germany, Agilent) [29]. The column temperature was fixed at 30 °C with a flow rate of 0.80 mL/min. Eluents A and B were used for gradient elution. Solution A was water with 0.1% phosphoric acid, and solution B was acetonitrile. The following gradient was used: 17% B (0 min), 15% (7 min), 20% (20 min), 24% (25 min), 30% (28 min), 40% (30 min), 50% (32 min), 70% (36 min) and 17% (40 min). For the analysis of phenolic compound fractions, the injection volume was 10 µL. Phenolic compounds were identified according to the retention times of the available pure compounds and the UV–Vis data obtained from authentic standards. Detection was carried out at 280, 320 and 360 nm. Concentrations are expressed as μg/mL. The results for phenolic compounds were the average of the analyses of three samples (*n* = 3).

### 2.5. Determination of Amino Acids

Amino acid content was determined via a method described by Bilgin et al. (2019) with slight modifications [30]. An amino acid analysis was performed using an LC system (Agilent Technologies, Waldbronn, Germany). MS/MS analyses were conducted on an Agilent 6460 triple quadruple LC–MS equipped with an electrospray ionization interface. The JASEM quantitative amino acids kit protocol (Sem Laboratuvar Cihazları A. Ş, Istanbul, Turkey) was used to determine amino acid compositions. The samples were read in the device after filtering without acidic hydrolysis and dilution. The results are expressed in mg/100 mL.

### 2.6. Color and Physicochemical Analyses

Brix was measured at 20 °C using an optical refractometer (ATAGO brand RX-7000α model, Japan), and pH was measured with a pH meter (Hanna Instruments HI 2002 pH/ORP, Romania). The titration acidity was potentiometrically determined via titration of the samples with a 0.1 N NaOH (Sigma-Aldrich, Burlington, MA, USA) solution to a pH of 8.1. An amount of 5 mL was taken from the sample, 50 mL of distilled water was added, and 10 mL of the sample was taken from the filtrate. The results were calculated as g tartaric acid/L.

The fruit juices’ L, a and b values were measured with a Hunter colorimeter (Color Measuring Device PCE-CSM 5, Marl, Germany). L is a measure of light and darkness in a range of 0–100. The number 0 corresponds to black, and 100 corresponds to white. In the color measurement system, the parameter’s positive (+) values indicate redness, and negative (-) values indicate greenness. The positive (+) values of the b parameter indicate yellow, and the negative (-) values represent blue. Chroma (C), hue angle (h), total color change (ΔE) and browning index (BI) are expressed according to the following Equations (2)–(6);

Chroma, C = (a^2^ + b^2^)^1/2^ (2)

h (hue angle) = tan^−1^(b/a) (3)

ΔE = ((ΔL)^2^ + (Δa)^2^ + (Δb)^2^)^1/2^ (4)

BI = [100(x − 0.31)] 0.17 (5)

where

x = (a* + 1.75L*)(5.645L* + a* − 3.012b*) (6)

All determinations were carried out three times per treatment.

### 2.7. Determination of Microbial Quality

Tests were performed to determine the current microbial load of freshly squeezed verjuice, including the enumeration of total coliform, total mesophilic aerobic bacteria, yeasts and molds. The Pour plate method was used to determine total mesophilic aerobic bacteria and coliforms, with plate count agar (PCA, Merck, Darmstadt, Germany), acidified (pH 3) Dichloran Rose Bengal Chloramphenicol (DRBC) and violet red bile agar (VRBA, Merck, Darmstadt, Germany) used to enumerate the total mesophilic aerobic bacteria, yeast–mold and total coliforms, respectively. PCA and VRB plate agars were incubated at 35 °C for 48 h and 35 °C for 24 h, respectively. The yeast and mold analysis was performed by spreading 0.1 mL on DRBC agar at 25 °C for five days. Results are given as CFU/mL.

### 2.8. Sensory Evaluation

The C-VJ, P-VJ and TS-VJ were presented to panelists who were asked to describe the differences between samples using a 9-point hedonic scale, where 1 is very much disliked, and 9 is very much liked. The sensory evaluation was based on the method of Yıkmış (2022) with slight modifications using 30 trained panelists (16 males and 14 females, from 20 to 25 years old) from Istanbul Esenyurt University who had received sensory assessment training [11]. The panelists participated in the sensory assessment by evaluating colors, smell, taste and overall acceptability features.

### 2.9. Statistical Analysis

All assays were performed in triplicate, and the results are expressed as mean ± standard deviation (SD). Data (C-VJ, P-VJ and TS-VJ) were analyzed by performing a one-way analysis of variance (ANOVA) (*p* < 0.05). The statistical analysis was conducted using SPSS 22.0 software (SPSS Inc., Chicago, IL, USA) and SigmaPlot 12.0 Statistical Analysis Software (Systat Software, Inc., San Jose, CA, USA).

## 3. Results

### 3.1. Optimization of Bioactive Components

The experimental and predicted results of the thermosonication effects applied to the bioactive components of freshly squeezed verjuice samples are given in Table 1. The experimental data of the study were analyzed using a second-order polynomial regression model. The quadratic–polynomial regression equations of the bioactive components resulting from the RSM modeling are shown below.

(7) $$TPC(mg\;GAE/100\;mL)=229.3+1.42X_{1}-40.86X_{2}+1.79X_{3}-0.11627X_{1}^{2}-\;0.6359X_{2}^{2}-0.09205X_{3}^{2}+0.4541X_{1}X_{2}+0.1016X_{1}X_{3}+0.6042X_{2}X_{3}$$

(8) $$TFC(mg\;CE/100\;mL)=-27.1+9.518X_{1}-35.84X_{2}+1.829X_{3}-0.11347X_{1}^{2}-\;0.3911X_{2}^{2}-0.02762X_{3}^{2}+0.3161X_{1}X_{2}-0.0570X_{1}X_{3}+0.5690X_{2}X_{3}$$

(9) $$DPPH\left(\%\;inhibition\right)=-13.8+5.789X_{1}-21.58X_{2}+1.028X_{3}-0.07038X_{1}^{2}-\;0.2416X_{2}^{2}-0.01689X_{3}^{2}+0.1881X_{1}X_{2}-0.03198X_{1}X_{3}+0.3457X_{2}X_{3}$$

(10) $$CUPRAC\left(\%\;inhibition\right)=-14.5+6.309X_{1}+24.07X_{2}-1.140X_{3}-0.07517X_{1}^{2}1-\;0.2567X_{2}^{2}-0.01765X_{3}^{2}+0.2118X_{1}X_{2}-0.03821X_{1}X_{3}+0.3812X_{2}X_{3}$$

**Figure 1:**
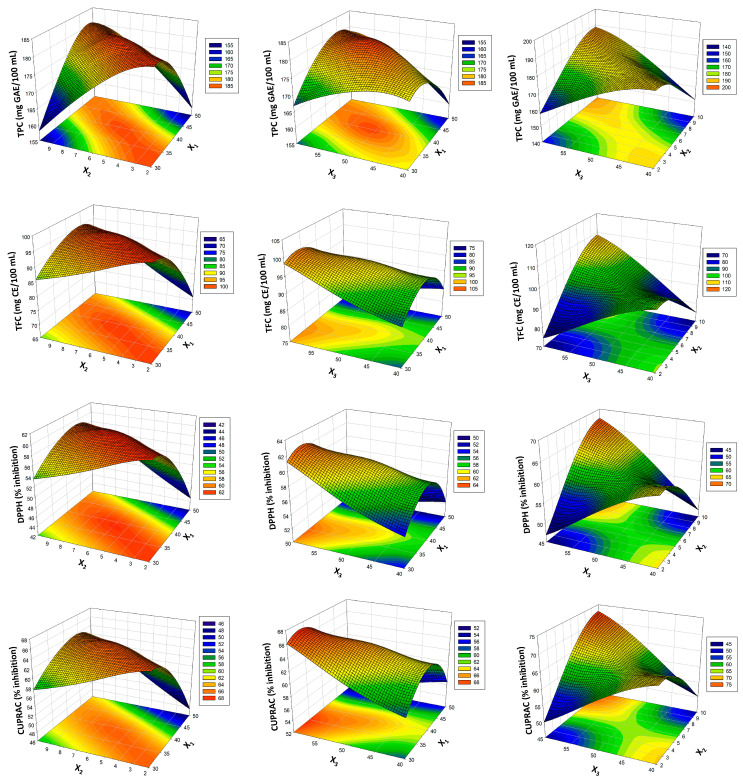
Response surface plots (3D) for bioactive compound analysis as a function of significant interaction factors for RSM.

Table 2 shows the analysis of variance (ANOVA) for TPC, TFC, DPPH and CUPRAC. While determining the optimization estimation height, model fit tests were performed for the TPC, TFC and antioxidant activity values of thermosonication-independent factors. Standard deviation, R^2^, adjusted R^2^ and predicted R^2^ values were considered for each function. According to these results, the R^2^ values of the bioactive components were determined as 98.39%, 00.23%, 99.30% and 99.20%, respectively. The R^2^ values of the results were >98%, proving that the predictive ability of the modeling is high. As seen in Table 2 for bioactive values, the quadratic (2nd order) function was found to be statistically significant (*p* < 0.05). It also showed similar properties in two-way interactions (*p* < 0.01). In addition, the temperature and time parameters for TPC were found to be statistically insignificant (*p* > 0.05). Three-dimensional RSM graphs with the determination of the best functions modeling the system are shown in Figure 1. When the figures of the bioactive components were examined, they showed an effective feature due to high predictive values. The parameters of the optimizations of the thermosonication process were determined as 41 °C, 10 min and 60 amplitude for X_1_, X_2_ and X_3_, respectively. As a result of the best thermosonication, its bioactive components can be enriched. TPC, TFC, DPPH and CUPRAC values were determined as 193.94 mg GAE/100 mL, 115.99 mg CE/100 mL, 72.00% inhibition and 77.81% inhibition, respectively. Similar increases in bioactive components were detected in thermosonication processes in kiwi peel [31] and freshly squeezed pomegranate juice treatments [11]. As can be seen in Table 1, as a result of the optimization of the study, it was determined that there was a high similarity when the actual values of the bioactive values of verjuice were compared with the estimated value. C-VJ, P-VJ and TS-VJ were also compared in the study. The thermal pasteurization process caused decreases in the bioactive components of verjuice. Similarly, it was found in many reports that the thermosonication process preserves or enriches bioactive components better than thermal pasteurization [12,32]. The reason for the enrichment or better preservation of bioactive components is that thermosonication processes enhance bioactive release and levels. The destruction of cell walls together with acoustic cavitation can be attributed to facilitate the release of bound compounds.

### 3.2. Amino Acids

Primary metabolites, such as amino acids, sugars, organic acids and fruit-specialized secondary metabolites, have a unique chemical structure that plays a role in the general functions of cells, giving an aroma and taste to fruit juices [33]. The amino acid profile and content provide valuable information about the quality, botanical origin and nutritional information of fruits and fruit-derived foods. As mentioned in the literature review [30], amino acid profiles also provide knowledge about adulteration assessments in commercial fruit juices. The total concentration of amino acids and proline may indicate the addition of sugar syrup or dilution with water to commercial juices [34].

This study examined 20 free amino acids in C-VJ, P-VJ and TS-VJ samples. A total of 17 free amino acids in various concentrations were identified, but glycine, taurine and cystine were not detected in any samples (Table 3). It was underlined that all the determined free amino acid values had statistical significance (*p* < 0.05) among the C-VJ, P-VJ and TS-VJ samples; only methionine levels showed no significant difference. Methionine values in our study (0.05–0.06 g/100 mL) were found to be lower than those of the data reported for the African pear pulp (0.81 g/100 g) sample [35]. There were no significant differences (*p* < 0.05) between the control and thermosonicated samples for the leucine and alanine contents.

The results of this investigation show that the amino acid contents with the lowest value to the highest value were 0.05 and 2.11 mg/100 mL for valine, tyrosine, threonine, leucine, isoleucine, glutamic acid and aspartic acid, which were detected at the highest rate in C-VJ. In contrast, arginine, histidine, lysine, ornithine and serine were detected at the highest level in TS-VJ samples. After the ultrasound, the decreased aspartic acid and glutamic acid results were similar to those of Erdal et al. (2002). In contrast, the alanine level did not significantly differ from C-VJ [36]. The control and ornithine samples were higher than the TS-VJ samples. The result of alanine in our study was lower than that of the reported mallow vinegar samples [37].

Arginine, an example of a neurotransmitter that plays an important role in wound healing, cell division and hormone-releasing [38], is obtained abundantly (2.11 ± 0.00 mg/100 mL) from thermosonication treatment. In total, 17 identified amino acids in P-VJ showed the lowest amino acid contents among C-VJ, TS-VJ and P-VJ. Among the 17 detected amino acids, leucine had the maximum value in C-VJ (0.81 mg/100 mL) and TS-VJ (0.79 mg/100 mL), followed by threonine (0.60 mg/100 mL) in C-VJ and histidine (0.60 mg/100 mL) in TS-VJ. The total concentration of essential amino acids was the highest at 3.75 ± 0.02 in C-VJ, followed by 3.52 ± 0.04 in TS-VJ and 1.95 ± 0.00 in P-VJ. Similarly, the results show that the total free amino acid contents were the highest (10.91 ± 0.02 mg/100 mL) in C-VJ, followed by TS-VJ (7.16 ± 0.02 mg/100 mL) and P-VJ (5.91 ± 0.00). The arginine levels were 29% and 18% of the total amino acid contents for the TS-VJ and C-VJ samples, respectively.

Amino acids can be affected by heat treatment, and lysine is particularly vulnerable to heat damage [39]. In this study, the lysine values were found to be decreased in the P-VJ sample compared to the C-VJ and TS-VJ samples. Amino acids are also precursors of volatile compounds that influence the juice aroma. In particular, leucine, isoleucine, valine and phenylalanine are involved in determining the aroma profile [40]. In this study phenylalanine, leucine, isoleucine and valine levels were obtained lower in P-VJ than in C-VJ and TS-VJ.

In contrast to Ahmed et al.’s findings (2019), which showed significantly enhanced free amino acid levels after thermosonication treatment of 30 °C for 20 min in wheat plantlet juice [41], we obtained significantly increased values in five amino acids at 41 °C for 10 min. Previous research has indicated potential associations between ultrasound treatment and the quality of proteins. Cavitational and mechanical stress resulting from ultrasound yields better extraction and functionalities of plant proteins [42]. Ahmed et al. [41] stated that higher free amino acid levels resulting from ultrasound treatment could rupture cell tissues, leading to the release of some free amino acids, and that heat treatment can modify amino acids when proteins react with different components (such as side chain degradation, denaturation and decreased amino acids that yield new links among amino acid interactions). Additionally, it has been suggested that the partial cleavage of intermolecular hydrophobic reactions can improve the release of free amino acids from ultrasound treatment [43].

Overall, the results of this study indicate that thermosonication at 41 °C for 10 min with 60 amplitude results in higher amino acid content compared to pasteurization treatment. The use of thermosonication under certain conditions in fruit juice processing could be an alternative method for increasing the total amino acid content and certain levels of free amino acids, such as arginine, histidine, lysine and others. However, the mechanism underlying the increase in free amino acids and its effect on fruit juices needs to be further researched.

### 3.3. Microbial Load

Tests were performed to determine the current microbial load of C-VJ, P-VJ and TS-VJ, including the enumeration of coliform, mesophilic aerobic bacteria, yeasts and molds. The Pour plate method was used to determine total mesophilic aerobic bacteria and coliforms, with plate count agar (PCA, Merck, Darmstadt, Germany), acidified (pH 3) Dichloran Rose Bengal Chloramphenicol (DRBC) and violet red bile agar (VRBA, Merck, Darmstadt, Germany) used to enumerate total mesophilic aerobic bacteria, yeast–mold and total coliforms, respectively. PCA and VRB plate agars were incubated at 35 °C for 48 h and 35 °C for 24 h, respectively. The yeast and mold analysis was performed by spreading 0.1 mL on DRBC agar at 25 °C for five days. Results are given as CFU/mL.

The results of this study indicate that freshly squeezed verjuice in laboratory conditions had no background microbial flora, as previously studied by Kaya and Unluturk (2019) and Kaya et al. (2020) [44,45]. P-VJ and TS-VJ samples also showed no microbial load of texted parameters.

Verjuice naturally has a self-protective system against *E. coli* and *S. typhimurium* pathogens and is widely used as an acidifying and flavoring agent in the food industry [46,47]. Although the antimicrobial activity of verjuice mainly depends on its organic acid content, its phenolic content is also thought to have an antibacterial effect against foodborne pathogens [5]. 

Verjuice is microbiologically safe due to its acidity and natural antimicrobial and antioxidant capacity [46]. However, verjuice and its products can be subject to spoilage via post-contamination [5]. As shown in their study, Karabiyikli and Oncül (2016) found that only one out of ten samples have no total mesophilic aerobic bacteria, coliforms, yeast or mold. Only one unripe grape sauce obtained from a local producer was more likely to be post-contaminated and showed initial microbial flora. Consequently, verjuice is more likely to be contaminated with yeasts under household production. Ultrasound treatment, approved by the FDA and which can reduce the microbial load or pathogens by 5 logs in fruit juices, can be used to ensure microbial safety and quality for verjuice products [5].

### 3.4. Phenolic Compounds

Phenolic compounds, which have a nutritive and antioxidant range, contribute to the taste and aroma of many plant-derived foods by affecting many sensory properties, such as flavor, astringency and color [48]. Phenolic samples (PCs) are phytochemicals with numerous bioactive properties found in many plants, including fruits. Although there are no nutrients in phenolic devices, their dietary intake provides protective effects for health [49]. In addition, they have anti-inflammatory, antipathogenic, antiallergic, antidiabetic, antiviral, antimicrobial and antithrombotic effects on human health [50].

Ripe or sour grapes have rich phenolic content. The total amount of phenolic substances in verjuice and juice samples from different regions has been stated to vary between 200 and 7538 mg GAE/L [1,51,52]. This study examined 13 phenolic filters in C-VJ, P-VJ and TS-VJ samples. Eight phenolic donors with various abilities were detected in the C-VJ sample, along with nine phenolic acceptors in the P-VJ sample and eleven phenolic contents in the TS-VJ sample. The content of phenolic products in the TS-VJ sample was increased by 37.5% according to C-VJ techniques and by 22.22% according to P-VJ techniques (Table 4). Nikfardjam (2008) stated that caftaric acid, catechin, epicatechin, fertaric acid, p-coutaric acid, protocatechuic acid and quercetin glycoside were detected in most verjuice samples. In contrast, quercetin, gallic acid, caffeic acid and coumaric acid could not be detected in these situations. The amount of these phenolic compounds, which have antioxidant properties, can change with the separation, filtration and thermal disruption effects in the process and may affect the final product range [53].

This study showed no statistical difference among the C-VJ, P-VJ and TS-VJ samples regarding ascorbic acid, gallic acid, protocatechuic acid, coumarin and vanillic acid contents. Ascorbic acid (vitamin C) has therapeutic and antioxidant properties. Of the vitamin C in the diet, 90% is provided by fruits and vegetables [54]. Immature fruits contain higher levels of vitamin C than mature fruits [55]. It has been determined that the vitamin C in groves obtained from different grape varieties varies between 15 and 20 mg/L [1,56]. The presence of oxygen and heat application are effective in the degradation of ascorbic acid [57]. Nayak et al. (2020) found a significant (*p* < 0.05) decrease in ascorbic acid content in pasteurized samples according to the level of ascorbic acid in raw elephant apple juice [58]. The ascorbic acid level was found to be 6.24  ±  0.21 g/L in raw fruit juice and 5.08  ±  0.18 g/L in pasteurized fruit juice. The decrease in ascorbic acid content can be explained by the heat sensitivity of ascorbic acid [59]. In his study, the thermosonication process inactivated watercress peroxidase under less severe bleaching conditions, and as a result, it was determined that the thermosonication process is a better blanching process because it keeps the vitamin C content at higher levels. The increase in ascorbic acid levels as a result of the thermosonication process may be due to the elimination of trapped oxygen molecules from the juice [60]. Whereas no statistical difference was observed between the C-VJ and P-VJ samples in catechin and hydroxybenzoic acid, a statistical difference was observed in the TS-VJ sample (*p* < 0.05). Ferulic acid, naringin and quercetin were undetectable in the C-VJ and P-VJ samples but were released in the TS-VJ samples. Although trans-cinnamic acid and o-coumaric acid were not detectable in the C-VJ and TS-VJ samples, they were revealed in the P-VJ samples. There was a statistical difference between the values detected in the neohesperidin C-VJ and TS-VJ samples (*p* < 0.05). Neohesperidin was not detected in the P-VJ sample (Table 4).

Yıkmış (2020) found an increase in the total phenolic concentration and total flavonoid concentrations in ultrasound-treated juice samples [61]. With the sonication process applied for 16 min, the total phenolic content of red watermelon juice increased by 1.7% compared to unprocessed red watermelon juice sample and by 5.7% compared to untreated yellow watermelon juice. In their study, Bhat et al. (2011) applied sonication to freshly squeezed Kasturi lime juice for 0, 30 and 60 min at 20 °C and 25 kHz [62]. They found that the concentration of the bioactive compound increased in most of the samples sonicated for 60 min compared to those treated for 30 min. This increase in total phenolic compound content can be explained by the cavitation pressure formed during the ultrasound process, which breaks down the cell wall and the bound form of the phenolic content, and by the binding of the hydroxyl radicals (·OH) produced by ultrasound to the aromatic ring of the phenolic compounds [57].

### 3.5. Physicochemical Properties and Color

The effects of the thermosonication and pasteurization processes applied to the verjuice samples on pH, brix, titratable acidity and color properties are presented in Table 5. Whereas a slight decrease was detected in the pH of verjuice after the pasteurization process (from 2.74 to 2.71), there was no change in the pH value after the thermosonication process. Similar results regarding pH have been reported in studies performed on pasteurized verjuice samples [63]; yellow and red watermelon juice samples [61]; thermosonicated purple cactus pear juices [64], carrot juice [65] and black mulberry juice samples [66]; and apple juice [21] samples. A slight decrease in pH was also reported in cashew apple nectar samples that were thermosonicated in a study conducted by Deli et al. (2022) [67].

However, no statistically significant change was observed in TSS and TA values after the pasteurization and thermosonication processes (*p* > 0.05). In their study, Deli et al. (2022) reported a similar result regarding TSS in thermosonicated cashew apple juice samples, and an increase in TA value was reported [67]. In other studies, TA and TSS values did not change in thermosonicated purple cactus pear juices [64], carrot juice [65] and black mulberry juice [66] samples. Ergezer et al. (2018) reported similar results regarding TA and TSS in pasteurized verjuice in their study, and Yıkmış (2020) reported similar results in yellow and red watermelon juice samples [61,63].

When the effect of the pasteurization and thermosonication processes on color was examined, there was no statistically significant difference in L (brightness–darkness), a (redness–greenness) and h values. In other words, the applied pasteurization and thermosonication processes did not change the L, a and h values of the color. However, b (yellowness–blueness) and C values decreased due to the operations. It has been observed that the pasteurization process reduces the b and C values more than the thermosonication process. Many studies have shown that thermosonication processes affect color parameters differently. In the study conducted by Cruz-Cansino et al. (2015), the thermosonication process decreased the L value in purple cactus pear juice samples and increased the a and b values [64]. In some studies on apple juice, black mulberry juice and carrot juice samples, the L, a and b values increased [21,65,66], along with reports of increases in C values in carrot juice samples [65]. In the study conducted by Herceg et al. (2013) on strawberry juice samples, L and a values decreased differently, and a similar decrease in the b value was reported [68]. Tomadoni et al. (2017) stated that the decrease in color values may be due to the increase in the amount of phenolic substances [69]. In this study, although the phenolic content increased, no change was observed in other parameters except for the increase in the C value and the decrease in the h value.

### 3.6. Sensory Analyses

**Figure 2:**
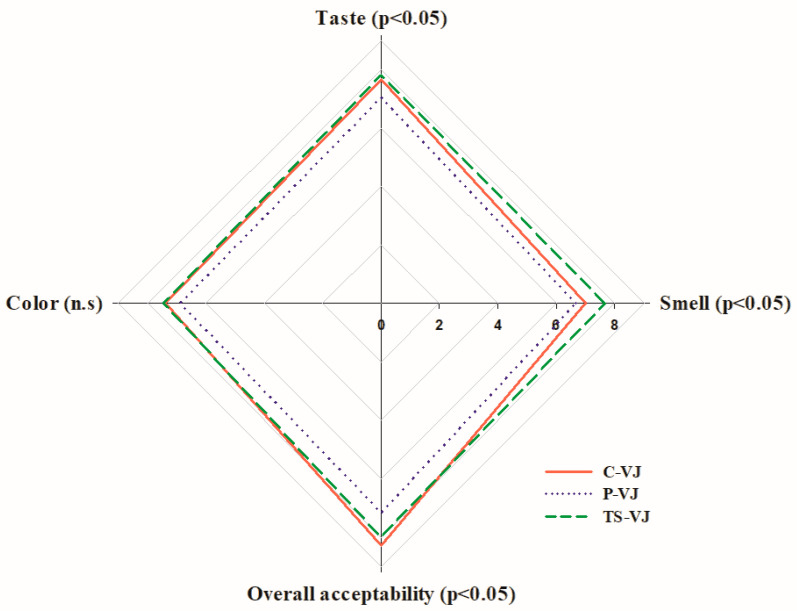
Sensory analysis results of C-VJ, P-VJ and TS-VJ samples.

The sensory analysis results evaluating color, smell, taste and general acceptability are given in Figure 2. When the samples were evaluated in terms of smell, one of the sensory analysis parameters, it was observed that the TS-VJ sample had the highest admiration score. There was no statistically significant difference between the C-VJ and P-VJ samples (*p* < 0.05). Statistically significant differences (*p* < 0.05) were found among the TS-VJ, C-VJ and P-VJ samples. When evaluated in terms of taste, whereas there was no significant difference between the C-VJ sample and the P-VJ and TS-VJ samples (*p* > 0.05), a significant difference was detected between the P-VJ and TS-VJ samples (*p* < 0.05). Regarding color parameters, no significant difference was found among the C-VJ, P-VJ and TS-VJ samples (*p* > 0.05). When the three samples were examined in terms of general acceptability, no significant difference (*p* < 0.05) was found between C-VJ (5.81 ± 1.46) and TS-VJ (8 ± 0.49b). It was found that the generally acceptable scores of the C-VJ and TS-VJ samples were significantly higher than that of the P-VJ sample. In a study with cashew apple juice, no significant difference was found between the sensory properties of thermosonicated and control samples [67]. In a study where pasteurization and thermosonication processes were applied to quince juice, Yıkmış et al. (2019) reported that there was no significant difference in color and general acceptability evaluation, and no significant difference was found between the two processes [70]. In the evaluation of taste, the pasteurized sample showed no significant difference, and the study found that the thermosonicated sample was the most popular in taste.

In conclusion, it was seen that the TS-VJ sample received the highest scores for smell (7.67), taste (7.83) and color (7.44). The thermosonication process was highly appreciated compared to the pasteurized sample in terms of sensory analysis, with organoleptic properties similar to freshly squeezed verjuice juice. The significant difference in the smell score from the C-VJ sample indicates that the thermosonication process can be used as an alternative to pasteurization. Anaya-Esparza et al. (2017) also stated that the thermosonication process in fruit juices can successfully increase enzymatic and microbial inactivation without causing a change in quality properties [10].

## 4. Conclusions

With this study, significant effects of thermosonication compared to thermal pasteurization were revealed. By optimizing the process conditions with RSM, bioactive components were increased. The use of thermosonication under specific conditions in fruit juices may increase the total number of amino acids and certain free amino acids, such as arginine, histidine and lysine. It can be used as an alternative method to pasteurization. However, further research is needed to investigate the mechanism of increasing free amino acids via thermosonication treatment and its effects. The thermosonication process did not cause significant changes in the physicochemical, color and sensory properties of verjuice. The thermosonication process caused increases in some phenolic compounds of verjuice. Thermosonication has been found to be a good alternative processing technology for thermal pasteurization. Pilot-scale studies are needed to better understand the possible effects of thermosonication. Moreover, more research is needed to understand the health-related relationships of thermosonication effects with in vivo studies.

## Figures and Tables

**Table 1:** Experimental and predicted responses of RSM and results of TS-VJ, C-VJ and P-VJ.

Sample	Encoded Independent Variables			Dependent Variables							
	Temputure (X_1_)	Time (X_2_)	Amplitude (X_3_)	TPC (mg GAE/100 mL)		TFC (mg CE/100 mL)		DPPH (% inhibition)		CUPRAC (% inhibition)	
				Experimental Data	RSM Predicted	Experimental Data	RSM Predicted	Experimental Data	RSM Predicted	Experimental Data	RSM Predicted
1	50	6	50	173.66 ± 2.67	172.44	81.57 ± 1.16	81.44	50.83 ± 0.86	50.89	54.65 ± 0.61	54.61
2	45	4	55	169.95 ± 2.14	170.26	79.29 ± 1.47	79.86	50.12 ± 0.76	50.21	53.13 ± 1.09	53.53
3	35	8	55	178.27 ± 1.58	177.57	100.88 ± 0.33	101.47	62.86 ± 0.23	63.16	67.59 ± 0.16	68.03
4	35	4	45	190.20 ± 2.80	189.45	99.87 ± 1.41	100.69	62.23 ± 0.92	62.66	66.91 ± 0.18	67.49
5	45	8	45	170.70 ± 1.05	171.05	88.91 ± 1.26	89.27	55.40 ± 1.03	55.55	59.57 ± 0.62	59.86
6	45	8	55	190.50 ± 0.40	190.96	99.81 ± 0.89	99.81	62.19 ± 0.48	62.20	66.88 ± 0.49	66.91
7	45	4	45	174.11 ± 2.64	174.52	91.86 ± 1.12	92.08	57.24 ± 0.76	57.38	61.55 ± 0.33	61.72
8	40	6	50	182.94 ± 0.71	184.83	96.80 ± 0.52	97.91	60.32 ± 0.64	61.04	64.86 ± 0.13	65.57
9	35	8	45	168.42 ± 1.24	167.82	84.99 ± 0.78	85.23	52.95 ± 0.18	53.31	56.94 ± 1.16	57.16
10	35	4	55	175.68 ± 0.81	175.03	93.72 ± 0.54	94.17	58.39 ± 0.58	58.69	62.79 ± 0.62	63.12
11	40	6	50	183.75 ± 0.75	184.83	98.69 ± 0.21	97.91	61.50 ± 0.07	61.04	66.12 ± 0.61	65.57
12	40	6	50	184.79 ± 0.51	184.83	98.62 ± 0.76	97.91	61.45 ± 0.95	61.04	66.08 ± 0.48	65.57
13	40	6	50	185.48 ± 1.67	184.83	98.35 ± 0.30	97.91	61.28 ± 0.23	61.04	65.90 ± 1.07	65.57
14	40	6	40	172.98 ± 1.15	172.89	93.58 ± 0.91	93.14	58.31 ± 0.49	58.01	62.70 ± 0.31	62.47
15	40	2	50	175.20 ± 1.06	175.13	90.01 ± 1.12	89.41	56.09 ± 0.55	55.86	60.31 ± 0.24	59.94
16	40	6	60	178.52 ± 1.09	178.37	97.55 ± 0.24	97.16	60.78 ± 1.24	60.69	65.36 ± 1.13	65.15
17	30	6	50	172.99 ± 0.27	173.98	92.38 ± 1.31	91.70	57.56 ± 0.62	57.12	61.89 ± 1.09	61.50
18	40	6	50	186.40 ± 0.75	184.83	98.14 ± 0.97	97.91	61.15 ± 0.72	61.04	65.26 ± 0.57	65.57
19	40	6	50	186.41 ± 0.40	184.83	97.69 ± 0.71	97.91	60.87 ± 0.45	61.04	65.48 ± 1.32	65.57
20	40	10	50	174.36 ± 1.16	174.19	94.12 ± 0.61	93.90	58.65 ± 0.30	58.50	63.06 ± 0.88	62.99
TS-VJ	41	10	60	190.44 ± 0.93	193.94	113.77 ± 0.91	115.99	70.77 ± 0.44	72.00	76.17 ± 1.34	77.81
C-VJ				172.44 ± 0.31		106.22 ± 0.52		63.25 ± 0.52		69.48 ± 0.49	
P-VJ				164.33 ± 0.47		97.33 ± 0.47		62.11 ± 0.54		65.33 ± 0.3	

TPC: Total phenolic content; TFC: Total flavonoid content; GAE: Gallic acid equivalent; DDPH: Radical scavenging activity; CUPRAC: Cupric reducing antioxidant capacity; CE: Catechin equivalent; C-VJ: Untreated verjuice; P-VJ: Thermal pasteurized verjuice; TS-VJ: Thermosonication-treated verjuice; RSM: Response surface methodology. Results are presented as mean ± standard deviation (*n* = 3).

**Table 2:** Corresponding *p*-values of linear, interaction and quadratic terms of regression coefficients obtained by RSM of responses for TPC, TFC, DPPH and CUPRAC parameters.

Source	DF	TPC (mg GAE/100 mL)		TFC (mg CE/100 mL)		DPPH (% Inhibition)		CUPRAC (% Inhibition)	
		F-Value	*p*-Value	F-Value	*p*-Value	F-Value	*p*-Value	F-Value	*p*-Value
Model	9	68.07	0.0000	142.6	0.0000	157.71	0.0000	138.58	0.0000
Linear	3	7.6	0.0060	84.12	0.0000	93.19	0.0000	82.4	0.0000
X_1_	1	1.52	0.2460	187.82	0.0000	205.22	0.0000	183.98	0.0000
X_2_	1	0.59	0.4590	36.13	0.0000	36.65	0.0000	35.39	0.0000
X_3_	1	20.7	0.0010	28.41	0.0000	37.68	0.0000	27.83	0.0000
Square	3	81.27	0.0000	133.86	0.0000	153.13	0.0000	127.84	0.0000
X_1_^2^	1	144.5	0.0000	358.51	0.0000	410.59	0.0000	343.32	0.0000
X_2_^2^	1	110.64	0.0000	109.01	0.0000	123.91	0.0000	102.47	0.0000
X_3_^2^	1	90.56	0.0000	21.24	0.0010	23.64	0.0010	18.93	0.0010
2-Way Interaction	3	115.33	0.0000	209.81	0.0000	226.82	0.0000	205.51	0.0000
X_1_ * X_2_	1	112.22	0.0000	141.63	0.0000	149.25	0.0000	138.72	0.0000
X_1_ * X_3_	1	35.11	0.0000	28.82	0.0000	26.98	0.0000	28.23	0.0000
X_2_ * X_3_	1	198.65	0.0000	458.98	0.0000	504.22	0.0000	449.58	0.0000
Error	10								
Lack-of-Fit	5	0.47	0.7860	1.23	0.4130	0.93	0.5310	1.03	0.4870
Pure Error	5								
Total	19								
R^2^		98.39%		99.23%		99.30%		99.20%	
Adj R^2^		96.95%		98.53%		98.67%		98.49%	
Pred. R^2^		94.15%		96.19%		96.78%		96.27%	

X_1_: Temperature; X_2_: Time; X_3_: Amplitude; DF: Degrees of freedom; TPC: Total phenolic content; TFC: Total flavonoid content; DDPH: Radical scavenging activity; CUPRAC: Cupric reducing antioxidant capacity; GAE: Gallic acid equivalent; CE: Catechin equivalent; *: multiply

**Table 3:** Effects of C-VJ, P-VJ and TS-VJ on free amino acids (mg/100 mL).

Analyses		Samples		
		C-VJ	P-VJ	TS-VJ
Amino acid content (mg/100 mL)	Alanine	1.11 ± 0.00 ^a^	0.46 ± 0.00 ^b^	1.10 ± 0.00 ^a^
	Arginine	1.98 ± 0.00 ^b^	1.04 ± 0.01 ^c^	2.11 ± 0.01 ^a^
	Aspartic Acid	1.20 ± 0.00 ^a^	0.76 ± 0.01 ^c^	0.92 ± 0.00 ^b^
	Cystine	n.d	n.d	n.d
	Glutamic Acid	1.10 ± 0.00 ^a^	0.66 ± 0.00 ^c^	0.93 ± 0.00 ^b^
	Glycine	n.d	n.d	n.d
	Histidine	0.57 ± 0.00 ^b^	0.32 ± 0.00 ^c^	0.60 ± 0.01 ^a^
	Isoleucine	0.33 ± 0.00 ^a^	0.25 ± 0.00 ^c^	0.30 ± 0.01 ^b^
	Leucine	0.81 ± 0.00 ^a^	0.52 ± 0.03 ^b^	0.79 ± 0.01 ^a^
	Lysine	0.35 ± 0.00 ^b^	0.13 ± 0.00 ^c^	0.39 ± 0.00 ^a^
	Methionine	0.06 ± 0.00 ^a^	0.05 ± 0.00 ^a^	0.06 ± 0.00 ^a^
	Ornitine	0.31 ± 0.00 ^b^	0.25 ± 0.00 ^c^	0.39 ± 0.01 ^a^
	Phenylalanine	0.54 ± 0.00 ^a^	0.37 ± 0.00 ^b^	0.54 ± 0.00 ^a^
	Proline	0.38 ± 0.01 ^a^	0.13 ± 0.00 ^b^	0.39 ± 0.00 ^a^
	Serine	0.79 ± 0.00 ^b^	0.50 ± 0.00 ^c^	1.04 ± 0.00 ^a^
	Threonine	0.60 ± 0.01 ^a^	0.13 ± 0.00 ^c^	0.36 ± 0.00 ^b^
	Tyrosine	0.29 ± 0.00 ^a^	0.16 ± 0.00 ^c^	0.28 ± 0.00 ^b^
	Valine	0.49 ± 0.00 ^a^	0.18 ± 0.00 ^c^	0.48 ± 0.00 ^b^
	Taurine	n.d	n.d	n.d

Results are presented as mean ± standard deviation (*n* = 3). Values with different letters within a line are significantly different (*p* < 0.05). C-VJ: Untreated verjuice; P-VJ: Thermal pasteurized verjuice; TS-VJ: Thermosonication-treated verjuice; n.d: not detected.

**Table 4:** Results of phenolic compounds of C-VJ, P-VJ and TS-VJ samples.

Compounds	Formula	Samples (μg/mL)		
		C-VJ	P-VJ	TS-VJ
Ascorbic acid	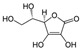	10.14 ± 0.16 ^a^	10.24 ± 0.31 ^a^	10.66 ± 0.97 ^a^
Gallic acid	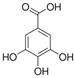	11.38 ± 0.41 ^a^	11.64 ± 0.44 ^a^	13.25 ± 1.42 ^a^
Protocatechuic acid	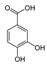	10.03 ± 0.30 ^a^	8.99 ± 0.64 ^a^	8.20 ± 0.81 ^a^
Catechin	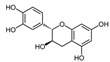	39.14 ± 1.55 ^a^	35.42 ± 1.76 ^a^	55.54 ± 3.27 ^b^
Hydroxybenzoic acid	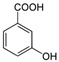	18.66 ± 0.62 ^a^	18.28 ± 1.03 ^a^	24.73 ± 1.32 ^b^
Vanillic acid	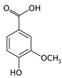	0.57 ± 0.11 ^a^	0.47 ± 0.04 ^a^	0.47 ± 0.05 ^a^
Ferulic acid	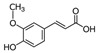	n.d	n.d	0.06 ± 0.01
Naringin	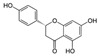	n.d	n.d	1.32 ± 0.26
o-coumaric acid	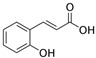	n.d	0.09 ± 0.01	n.d
Neohesperidin	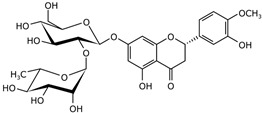	0.05 ± 0.03 ^a^	n.d	0.08 ± 0.02 ^b^
Coumarin	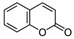	0.09 ± 0.00 ^a^	0.10 ± 0.02 ^a^	0.05 ± 0.02 ^a^
Quercetin	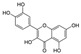	n.d	n.d	1.11 ± 0.17
Trans-cinnamic acid	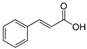	n.d	0.17 ± 0.03	n.d

Results are presented as mean ± standard deviation (*n* = 3). Values with different letters within a line are significantly different (*p* < 0.05). C-VJ: Untreated verjuice; P-VJ: Thermal pasteurized verjuice; TS-VJ: Thermosonication-treated verjuice; n.d: not detected.

**Table 5:** Analysis results of color, microbiological analyses and physicochemical parameters of C-VJ, P-VJ and TS-VJ samples.

Analyzes		Samples		
		C-VJ	P-VJ	TS-VJ
Physicochemical parameters	pH	2.74 ± 0.01 ^a^	2.71 ± 0.01 ^b^	2.74 ± 0.01 ^a^
	TSS (°Brix)	3.07 ± 0.03 ^a^	2.43 ± 1.16 ^a^	3.08 ± 0.03 ^a^
	TA (g TA/L)	3.83 ± 0.03 ^a^	3.80 ± 0.00 ^a^	3.83 ± 0.03 ^a^
Color properties	*L*	49.33 ± 1.27 ^a^	47.39 ± 1.92 ^a^	46.06 ± 0.49 ^a^
	*a*	4.44 ± 0.29 ^a^	3.94 ± 0.30 ^a^	3.96 ± 0.04 ^a^
	*b*	15.89 ± 0.15 ^a^	15.37 ± 0.10 ^b^	15.56 ± 0.06 ^b^
	Chroma (C)	16.50 ± 0.09 ^a^	15.87 ± 0.04 ^b^	16.05 ± 0.07 ^c^
	Hue angle (h°)	74.40 ± 1.08 ^a^	75.62 ± 1.10 ^a^	75.71 ± 0.10 ^a^
	BI	44.83 ± 1.45 ^a^	44.67 ± 2.26 ^a^	46.79 ± 0.42 ^a^
	ΔE	-	2.14 ± 0.69	3.34 ± 1.09
Microbiological analyses (log CFU/mL)	TMAB	n.d	n.d	n.d
	YM	n.d	n.d	n.d
	CB	n.d	n.d	n.d

Results are presented as mean ± standard deviation (*n* = 3). Values with different letters within a line are significantly different (*p* < 0.05). C-VJ: Untreated verjuice; P-VJ: Thermal pasteurized verjuice; TS-VJ: Thermosonication-treated verjuice; TA: titratable acidity; TSS: Total soluble solids; ΔE: Total color change; BI: Browning index; TMAB: Total mesophilic aerobic bacteria; YM: Yeasts and molds; CB: Coliform Bacteria; n.d: not detected.

## Data Availability

Data is contained within the article.
